# Oviposition Behavior of *Aedes aegypti* and *Aedes albopictus* (Diptera: Culicidae) from Panama Under Experimental L4-Larval Co-Occurrence Scenarios

**DOI:** 10.3390/insects16111110

**Published:** 2025-10-31

**Authors:** Reyna Tuñón, Mabelle Chong, Ambar L. Rojas, Armando Castillo, Callum Kingwell, Luis F. Chaves, Jose R. Loaiza

**Affiliations:** 1 División de Biodiversidad y Una Salud, Unidad de Vigilancia de Zoonosis, Instituto de Investigaciones Científicas y Servicios de Alta Tecnología (INDICASAT AIP), Panama City 0843-01103, Panamaamanita15chong@gmail.com (M.C.);; 2 Programa Centroamericano de Maestría en Entomología, Universidad de Panama, Campus Octavio Méndez Pereira, Avenida Transístmica, Panama City 0816-03366, Panama; lfchavs@gmail.com; 3 Centro de Neurociencias (INDICASAT AIP), Panama City 0843-01103, Panama; 4 Department of Ecology and Evolutionary Biology, Princeton University, Princeton, NJ 08544, USA; ck0938@princeton.edu; 5 Smithsonian Tropical Research Institute, Panama City 0843-03092, Panama; 6 Department of Environmental and Occupational Health, School of Public Health, Indiana University, Bloomington, IN 47405, USA; 7 Department of Geography, Indiana University, Bloomington, IN 47405, USA

**Keywords:** container-breeder, *Aedes aegypti*, *Aedes albopictus*, segregated oviposition, tropical Panama

## Abstract

While a great deal of research has been conducted to identify the biotic mechanisms influencing the co-occurrence of aquatic *Ae. aegypti* and *Ae. albopictus* mosquitoes, including inter-specific larval competition and mating interference, few studies have been completed to investigate their oviposition behaviors under co-occurrence scenarios. Herein, we performed three experiments to understand the influence of conspecific and heterospecific L4-larvae presence in oviposition by antagonist *Aedes* spp. mosquitoes. We compared egg counts of *Ae. aegypti* and *Ae. albopictus* in cups with water and conspecific or heterospecific larvae (either present or removed), or no larvae as control. In the first experiment, we used small cages; in the second experiment, we used larger cages increasing the number of treatment replicates per cage; and in the third experiment, we removed larvae before allowing gravid females to oviposit. In experiments 1 and 2, *Ae. aegypti* laid more eggs in conspecific cups, while *Ae. albopictus* distributed eggs evenly between conspecific and heterospecific cups, and both species laid fewer eggs in controls. In these experiments, more eggs were laid during the first 24 h. In experiment 3, both species laid more eggs in conspecific larvae-conditioned water as compared to controls in the first 24 h; however, in *Ae. Aegypti*, this inclination was lost with time. Our results suggest that co-occurrence of *Ae. aegypti* and *Ae. albopictus* may be explained by *Ae. aegypti*’s ability to avoid oviposition in habitats colonized by heterospecific L4-larvae. Yet chemical cues in conspecific L4-larvae-conditioned water were insufficient to replicate this behavior, and therefore, additional cues may be involved.

## Simple Summary

*Aedes aegypti* (Linnaeus) and *Aedes albopictus* (Skuse) are important global disease vectors. These mosquito species either coexist or replace each other spatially depending on the local environmental conditions, yet the ecological mechanisms that shape their demography in areas of spatial overlap are still poorly understood. We assess the oviposition behavior of *Ae. aegypti* and *Ae. albopictus* when presented with containers holding conspecific and heterospecific larvae. We show that gravid *Ae. aegypti* rapidly finds and lays eggs in conspecific containers, presumably to reduce the possibility of larval competition with *Ae. albopictus*. Nevertheless, in the absence of visual cues, *Ae. aegypti*’s capacity to discriminate between conspecific choices tends to deteriorate, resulting in a greater number of eggs being laid in heterospecific containers. Gravid *Ae. albopictus*, instead, does not exhibit a preference for either conspecific or heterospecific containers.

## 1. Introduction

The yellow fever mosquito, *Aedes* Stegomyia *aegypti* (Linnaeus), and the Asian tiger mosquito, *Aedes* Stegomyia *albopictus* (Skuse), are prominent insect pests owing to their ability to invade new geographic areas assisted by the transport of human commodities and their capacity to transmit life-threatening viral pathogens to humans [1,2,3,4]. These mosquitoes lay their eggs in natural and artificial water-holding containers along a gradient of forested, rural, and urban ecological conditions [3,4]. The larvae compete with one another for space and limited food resources in breeding sites of varying water quality [5,6], while males are able to mate with heterospecific females, resulting in various degrees of reproductive interference via bi-directional satyrization [7]. Due to their evolutionary, ecological, and demographic similarities, *Ae. aegypti* and *Ae. albopictus* now overlap in a significant portion of their globally expanded geographic ranges [8].

To date, there is a comprehensive body of literature concerning the competitive interaction between *Ae. aegypti* and *Ae. albopictus*, but efforts to understand the biotic factors that can shape their spatial population demography, other than larval competition and interspecific mating, are relatively scant. In particular, the distribution and competitive dynamics of these species might be influenced by the resource quality of container habitats [9,10,11,12] the outcome of interspecific mating interference [7], intrinsic differences in egg surface morphology affecting drought resilience [13], and environmental conditions such as regional variation in climate and urbanization [14,15,16,17]. Further, little is known about the oviposition behaviors of *Ae. aegypti* and *Ae. albopictus* under discrete larval habitat settings in the context of species co-occurrence [18,19,20].

Oviposition site selection has direct effects on mosquito relative fitness and population demography [21,22]. Gravid females of antagonist *Aedes* species may select egg-laying containers based on factors such as the presence of conspecific and heterospecific eggs or larvae [18,19,23,24], larval food availability [5,6,25], container characteristics (size, shape, material, color) [26,27,28,29], or volatile organic compounds acting as aggregating or segregating kairomones [30,31,32,33,34]. Visual cues are essential for the nocturnal host-seeking behavior of both *Ae. aegypti* and *Ae. albopictus* [35], while wing stroke records of *Ae. aegypti* indicated that carbon dioxide (CO_2_) is a visual attention enhancer for host-seeking females but not for gravid ones [36]. In multiple-choice tests, gravid *Ae. aegypti* prefer to oviposit in water conditioned with conspecific fourth-instar larvae over water conditioned with other larval stages [29]. Furthermore, gravid *Ae. aegypti* lay significantly more eggs in field traps loaded with L4-stage conspecific larval extracts plus *Bacillus thuringiensis israelensis* (Bti) than in control traps [31]. The effect of visual signals and olfactory cues, including CO_2_ and conspecific and heterospecific larval-related cues, in determining the oviposition performance of *Aedes* mosquitoes, is still largely unknown [33,37].

Previous studies suggest that *Ae. aegypti* may distribute eggs across multiple breeding containers during a single gonotrophic cycle. This strategy, known as “Skip Oviposition,” is thought to increase progeny survival by decreasing intraspecific larval competition, particularly in food-limited aquatic environments [21,38,39,40,41]; but, also, see the work by Harrington and Edman [42]. In contrast, other studies have proposed a different oviposition behavior whereby gravid *Ae. aegypti* lay the vast majority of eggs from the same gonotrophic cycle in a single container. This strategy, known as “Favorite Cup,” is thought to help gravid females conserve energy by reducing flight distance and time during oviposition-site searching [43]. Another possible explanation is that *Ae. aegypti* avoids laying eggs in containers where a superior larval competitor is already present, thereby reducing the risk of interspecific biological competition. Whether waterborne cues in discrete larval-conditioned containers (e.g., conspecific versus heterospecific) influence the oviposition performance of *Ae. aegypti* and *Ae. albopictus* remains uncertain [41,44]. Moreover, relatively little information is available on the adoption and performance of either “Skip Oviposition” or “Favorite Cup” strategies in *Aedes* mosquitoes under experimental scenarios of species co-occurrence [19,44,45].

Understanding egg-laying choices and their implications for *Aedes* species interaction may assist in predicting how populations of these mosquitoes will respond to traditional control measures [21,33]. For example, identifying key species-specific behaviors that aggregate or segregate oviposition in the presence of direct larval competitors could allow control methods to be tuned to disproportionately impact the species with higher vector competence. Such an approach may be a more cost-effective mode of vector control in areas of species co-occurrence than eliminating all existing larval containers indiscriminately [21,33]. From an epidemiological perspective, there is therefore a need to better understand whether *Ae. aegypti*, the principal vector of emergent Chikungunya and Zika viruses in tropical regions worldwide [1,2,8], chooses or avoids heterospecific containers as oviposition sites.

*Aedes aegypti* was initially reported in Panama in 1902, whereas *Ae. albopictus* was first detected a century later, in 2002 [46]. Since the arrival of *Ae. albopictus*, these two species have competed for space and resources across the country, with outcomes ranging from displacement to coexistence depending on the climatic, environmental, and socio-economic conditions in invaded settlements, as well as microbiome and genomic factors [15,16,47,48,49,50,51]. Field empirical data from tropical Panama further suggest that eggs from these mosquitoes are rarely found in the same oviposition container, even within areas of known species coexistence. Further, eggs of *Ae. aegypti* are more likely than those of *Ae. albopictus* to occur alone in oviposition traps [15,48]. Whether the two species actively avoid one another’s oviposition sites due to visual or non-visual olfactory recognition signals discrete to each species, or whether one species outcompetes the other in situ through interspecific biological antagonism, is yet to be investigated in Panama.

The goal of this study was to investigate the oviposition behavior of *Ae. aegypti* and *Ae. albopictus* in the presence of conspecific and heterospecific fourth instar (L4) larvae at oviposition sites. Under an experimental co-occurrence scenario, we hypothesize that gravid *Ae. aegypti* lay more eggs in containers with conspecific L4-larvae than in those with heterospecific larvae. This prediction follows the “Favorite Cup” pattern, in which a substantial proportion of eggs from a single gonotrophic cycle are oviposited in one container, likely to avoid interspecific competition with *Ae. albopictus* [42,43]. In contrast, under an experimental co-occurrence scenario, we hypothesize that gravid *Ae. albopictus* distribute eggs more evenly between conspecific and heterospecific choices. This prediction follows the “Skip Oviposition” pattern, in which eggs from a single gonotrophic cycle are partitioned among multiple containers, reducing intraspecific competition while increasing the likelihood of interspecific competition with *Ae. aegypti* [41,44,45]. This information is necessary for the effective deployment of targeted vector control tactics against gravid females of these two species in areas of Panama where they coexist.

## 2. Materials and Methods

### 2.1. Mosquito Sampling and Colony Establishment

The Azuero Peninsula (AP) is one of the few places in Central Panama where *Ae. aegypti* and *Ae. albopictus* still co-occur in some localities, with the latter having completely displaced the former in other localities during the last five years [15]. We gathered eggs of *Aedes* mosquitoes using oviposition traps (i.e., Ovitraps) and conducted active surveillance to gather larvae and pupae in natural and artificial containers around houses from two localities in AP. Parita, located in the northeastern part of AP (8°00′07.9″ N; −80°31′39.0″ W), having a warmer, drier, and less vegetated environment—with both *Ae. aegypti* and *Ae. albopictus* currently present—was selected to gather samples of *Ae. aegypti*. Tonosi, located in the southwestern part of AP (7°24′44.0″ N; −80°26′09.0″ W), having a cooler, wetter, and more vegetated environment—but with *Ae. aegypti* currently displaced—was selected to collect samples of *Ae. albopictus* [15,16]. Field samples were used to establish a colony of *Ae. aegypti* from Parita and another colony of *Ae. albopictus* from Tonosi.

Eggs were collected on filter papers, dried at room temperature, and stored in Petri dishes for one week inside cardboard envelops in an environmental chamber at 21 degrees Celsius (°C). Eggs were placed in 500-milliliter (mL) plastic rearing trays, submerged in dechlorinated tap water at 25 +/− 2 °C, and first-instar larvae were fed on commercial fish food TetraMin tropical flakes throughout the pupal stage in separate trays per species (TETRA, Blacksburg, VA, USA). Larvae were fed with 3.0 mL of TetraMin macerated solution (0.2 milligrams/mL) per larva during the first two days, 3.0 mL (0.3 mg/mL) per larva in the third day, and 3.0 mL (0.4 mg/mL) per larva on days 4 through 6. Pupae were transferred to 500 mL plastic cups and allowed to emerge as adults inside standard Bioquip 30 × 30 × 30-centimeter (cm) rearing entomological cages (Bioquip products Inc., Compton, CA, USA). Upon completing the aquatic developmental cycle in the laboratory, mosquitoes were identified taxonomically based on adult morphological characters [52]. An equal proportion of males and females of the F0 generations from an equal number of houses in Parita and Tonosi were selected and allowed to mate in order to build the corresponding F1 mosquito colonies of each species.

Immature stages (i.e., larvae and pupae) from the respective F1 generations of each mosquito species were reared to adulthood in a modular insectary at 25 +/− 2 °C, 80–90% relative humidity, and a photoperiod of 12:12 (Light: Dark) hours. At each subsequent generation, nulliparous females were separated from the colony and put inside a small cage along with five males from the same cohort, offered *ad libitum* 10% sucrose solution, and fed on human blood from a volunteer. Fully engorged, mated females were rested for 48 h in individual experimental cages prior to oviposition trials, which were conducted exclusively with gravid females that had fully digested their blood meal. Mosquito generations F1 through F3 of both *Ae. aegypti* and *Ae. albopictus* were used to establish the corresponding colonies. Only F3+ females were used in the oviposition trials to control for maternally derived phenotypic variation.

#### 2.1.1. Experiment # 1: Oviposition Choices of *Aedes* Species in Small Cages

**Figure 1:**
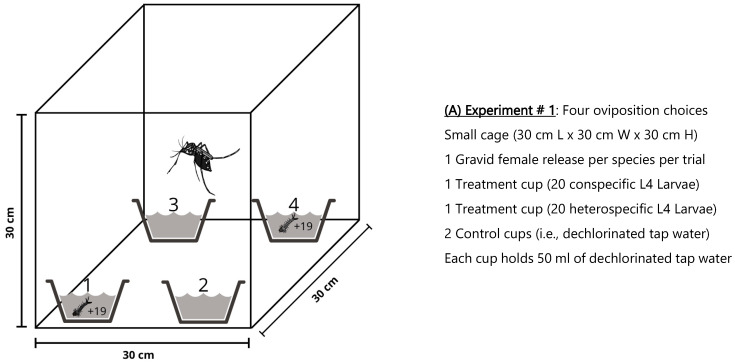

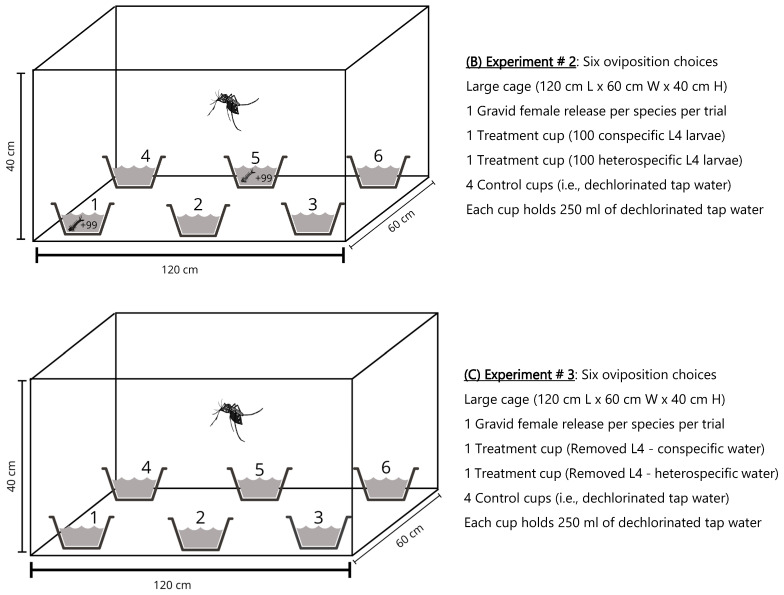
Diagram of oviposition experiments # 1 (**A**), # 2 (**B**), and # 3 (**C**).

We set up our first oviposition experiment inside standard Bioquip 30 cm L × 30 cm W × 30 cm H entomological cages (Figure 1A) (Bioquip products Inc., Compton, CA, USA). We establish four oviposition choices, including two treatments, and two controls within each experiment replicate. Treatments contained 20 L4-instar larvae of either *Ae. aegypti* or *Ae. albopictus*, each in disposable cardboard cups holding 50 mL of pre-filtered dechlorinated tap water, while the controls hold only 50 mL of pre-filtered dechlorinated tap water. Oviposition water was filtered using an under-sink Multi-Stage Filtration System to remove both contaminants and bacteria. In order to further prevent bacteria from growing in the oviposition water, the L4 larvae of both species were rinsed three times in pre-filtered water and given an hour to defecate before the tests began. We also used disposable Pasteur pipettes and gloves to transfer larvae to treatment cups, and autoclaved all non-disposable equipment between replicates to avoid bacterial contamination [9]. The position of each container inside the trial cage was randomized by assigning four places representing the corners of the cage, separated roughly at equal distance from each other and from the center. This allowed for impartial positioning of conspecific L4-larvae, heterospecific L4-larvae, and controls at each experimental replicate.

A sheet of Grade 1 Whatman^®^ qualitative filter paper (W × L 460 mm × 570 mm) was provided as oviposition substrate. The filter paper was cut in bands and wrapped upright around the edges and attached to the inside of each cup, such that half was exposed above the water line. We used the same white filter paper type as oviposition substrate for gravid females in all the containers. Experiment # 1 was conducted separately for *Ae. aegypti* and *Ae. albopictus*, with 60 replicates run per species, each corresponding to a single gravid female released in the cage and left inside for a period of 72 h. We selected this time period because the duration of the gonotrophic cycle in *Aedes* mosquitoes, which is the time between a blood-feeding event and oviposition, varies by species but is typically 3.7–4.2 days for *Ae. aegypti* and 3.2–3.7 days for *Ae. albopictus* [53]. At each replicate, eggs were counted three times, under 40× magnification, by the same observer to confirm the exact number, using a manual counter and a stereoscope LEICA model S9E (Leica Microsystems, Hollywood, FL, USA). We recorded the number of oviposited eggs by species, oviposition choice, and time. The number of eggs laid at 24, 48, and 72 h post-release was obtained by counting and subtracting the preexisting eggs from the total number of eggs present at each oviposition container (i.e., both in the papers and in the water) at those time periods [40].

#### 2.1.2. Experiment # 2: Oviposition Choices of *Aedes* Species in Large Cages

We set up our second oviposition experiment inside larger home-made 120 cm L × 60 cm W × 40 cm L cm entomological cages (Figure 1B). We added two additional controls to corroborate the initial oviposition behavior recorded for gravid *Ae. aegypti* and *Ae. albopictus* during the first experiment. This allowed us to better accommodate for the increasing biological complexity of the “Skip Oviposition” or “Favorite Cup” behaviors in *Aedes* mosquitoes, by not forcing gravid females to lay eggs in a limited number of containers inside a small experimental environment.

We presented six oviposition choices within each experimental replicate. Two oviposition sites contained either 100 L4-larvae of *Ae. aegypti* or *Ae. albopictus* in disposable cardboard cups with 250 mL of pre-filtered dechlorinated tap water, and four control sites containing only 250 mL of pre-filtered dechlorinated tap water. There were more larvae per container in this second experiment, but the proportion of larvae in the water volume (0.4 larvae per milliliter) was the same as in the first experiment, both of which had undercrowded conditions that are considered important for high-quality larval habitat [11,12]. The position of oviposition containers inside the trial cage was again randomized by assigning six places representing each corner plus two spots in the middle and lateral sides of the cage (Figure 1B). This allowed for impartial positioning of conspecific L4-larvae, heterospecific L4-larvae, and controls at each experimental replicate. As oviposition substrate for gravid females, we used the same Grade 1 Whatman^®^ qualitative filter paper type as in the first experiment in all the containers (Whatman-Sigma Aldrich, Rockville, MD, USA). Experiment # 2 was conducted separately for *Ae. aegypti* and *Ae. albopictus*, with 87 replicates run per species, corresponding to a single gravid female released in the cage and left inside for a period of 72 h. Eggs laid were counted both in the papers and in the water as explained in experiment # 1 [40].

#### 2.1.3. Experiment # 3: Oviposition Choices of *Aedes* Species in the Absence of L4-Larvae

We repeated all the steps from the second experiment, using the same water from oviposition choices (i.e., treatments and controls), but removing the L4-larvae of *Ae. aegypti* and the L4-larvae of *Ae. albopictus* from treatment cups (Figure 1C). We performed this test to investigate whether or not *Aedes* species can display the preferred oviposition behavior by discriminating between conspecific and heterospecific containers, but without L4-larvae as visual signals. If both species maintained the same oviposition behaviors as the ones displayed in the first two experiments, this could mean that non-visual olfactory cues, in addition to visual stimuli, could play an important role in regulating the species-specific oviposition choices in *Aedes* mosquitoes. Alternatively, if they change their oviposition behaviors compared with the first two experiments where treatments contained L4-larvae, then olfactory stimuli might be less crucial at shaping the species-specific oviposition choices in the presence of an antagonist species. We set up the third experiment within less than one hour upon finishing Experiment # 2 in order to keep L4-larval volatile organic compounds in the water, which may be used as attractive or repellent olfactory cues for gravid *Aedes* females. Experiment # 3 was conducted separately for *Ae. aegypti* and *Ae. albopictus*, with 24 replicates run per species, corresponding to a single gravid female released in the cage and left inside for a period of 72 h. Laid eggs were counted both in the papers and in the water as explained in experiment # 1 [40].

### 2.2. Metabolic Rates

We assessed carbon dioxide (CO_2_) production by L4 larvae *Ae. aegypti* and L4 larvae *Ae. albopictus* in order to pinpoint non-visual species-specific signals that might be used by gravid females to discriminate between conspecific and heterospecific cups. Although we used larvae that were extracted from the same mosquito colonies that provided samples for the oviposition experiments, these larvae were not directly used in the experiments. Larvae were individually removed from the holding containers with a pasture pipette and rinsed with dechlorinated tap water to eliminate excess debris. A group of 100 L4-larvae from each species were placed in separate 50 mL dechlorinated tap water solution beakers and transferred into 250 mL gas sampling bottles (i.e., Nalgene bottle with lid) encompassing a built-in adapter connected to the Vernier Labquest2 CO_2_ and oxygen (O_2_) sensors. We adjusted the sensors with the Vernier’s Logger Pro 3.16 software analysis system using default settings. Gaseous CO_2_ estimates were recorded using the Vernier CO_2_ Gas Sensor set up at low range (i.e., 0 to 10,000 ppm) for 20 min in each replicate, with readings taken every 2 s inside a plexiglass chamber at 25 °C (±5 °C). We employed default settings at each replicate measurement, including warm-up time of 90 s, gas sampling mode (i.e., diffusion), and typical accuracy at standard pressure (i.e., 1 atm). We conducted >50 replicate measurements for each of three following groupings: *Ae. aegypti* L4 larvae, *Ae. albopictus* L4 larvae, and control solutions (e.g., pure water that never held any larvae).

### 2.3. Statistical Analysis

We analyzed the results from each of the three oviposition experiments using Poisson Generalized Linear Mixed Effects Models—GLMM [54]. This modeling approach was chosen given the study design, where experiments were performed using assays, performed through time-given logistic limitations to do all replicates at the same time [55]. For this reason, in the mixed model, we include a random factor for “assay”, while time (e.g., 24, 48 and 72 h) and treatment (e.g., conspecific versus heterospecific containers) were the fixed factors. We also included a Poisson response, given the nature of the response variable which are egg counts. In all cases, we compared models that assumed time and treatment as independent, and also models accounting for an interaction between time and treatment, selecting the best model for each case based in the minimization of the Akaike Information Criterion (AIC), a common tool for model selection [56]. A one-way analysis of variance (ANOVA) with post hoc Tukey’s HSD test for multiple comparison was performed to compare the effect of three discrete groupings (i.e., L4-larvae of *Ae. aegypti*, L4-larvae of *Ae. albopictus*, and control solution) on the production of gaseous CO_2_, using the software package R (Version 4.5.1) [57].

## 3. Results

In total, we recovered 2789 eggs from gravid *Ae. aegypti* and 3117 eggs from gravid *Ae. albopictus*. Of these, 47.6%, 34.2%, and 18.2% were laid by *Ae. aegypti*, while 33.8%, 41.0%, and 25.6% were laid by *Ae. albopictus* during the first, second, and third oviposition experiments, respectively. Overall, conspecific containers consistently produced more eggs per container treatment in both *Ae. aegypti* and *Ae. albopictus* compared with heterospecific containers and controls (Table 1). The mean number of eggs laid (±SD) in conspecific containers was always larger for *Ae. aegypti* than for *Ae. albopictus*, except during the third experiment when L4-larvae were removed from treatments (Table 1). Conversely, the mean number of eggs laid (±SD) in heterospecific containers was always larger for *Ae. albopictus* than for *Ae. aegypti* in all three experiments (Table 1). The mean number of eggs laid (±SD) in controls was larger for *Ae. aegypti* during the first experiment, but more eggs were laid on average by *Ae. albopictus* in controls during the second and third experiments (Table 1). The GLMM results for the three experiments with gravid *Ae. aegypti* and gravid *Ae. albopictus* are shown in Table 2 and Table 3, respectively. The GLMMs assuming that treatment and time interact with each other were a better fit to the data than the GLMMs assuming the independence of treatment and time, except in the third experiment with gravid *Ae. aegypti* (Table 2).

We detected a statistically significant difference in the mean CO_2_ production between at least two groups (F(2, 162) = [32.81], *p* = 1.09 × 10^–12^). Mean CO_2_ production was significantly different between *Ae. aegypti* L4-larvae and the control (*p* < 0.0001, 95% C.I. = [−0.054, −0.028]) and between *Ae. albopictus* L4-larvae and the control (*p* < 0.0001, 95% C.I. = [−0.049, −0.023]). However, there was no statistically significant difference in the mean CO_2_ production between L4-larvae of *Ae. aegypti* and L4-larvae of *Ae. albopictus* (*p* = 0.658) (Figure S1).

### 3.1. Oviposition Experiment # 1 with Gravid Aedes aegypti

**Figure 2:**
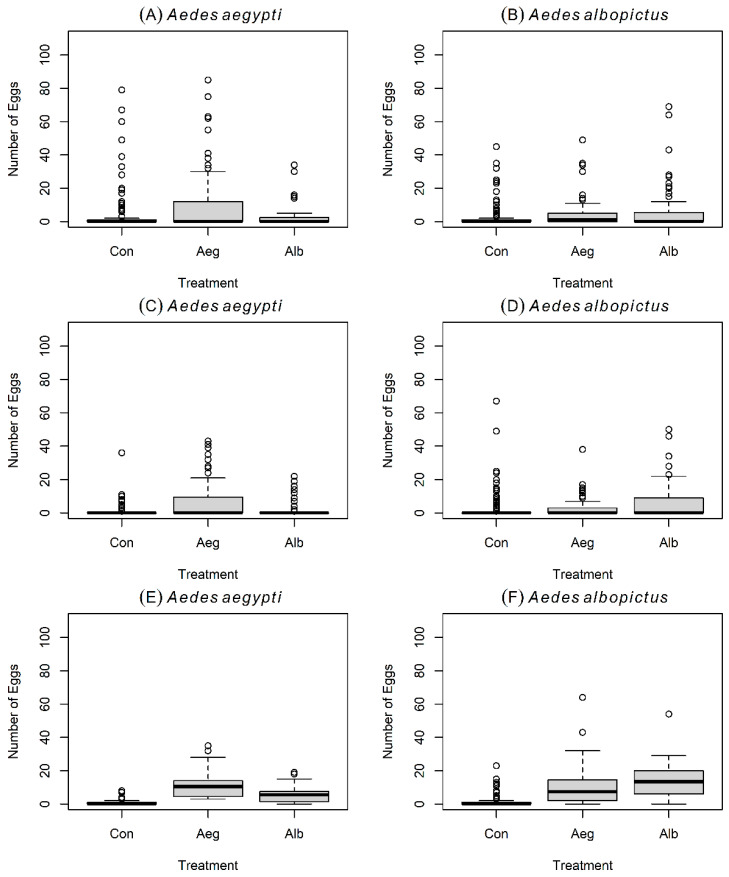
Boxplots (median and quantiles) of eggs laid by gravid *Aedes* spp. mosquitoes in the three experiments. Results of Experiment 1 (=small entomological cages with larvae) are shown for gravid *Ae. aegypti* and for gravid *Ae. albopictus* in (**A**,**B**) in that order. Results of Experiment 2 (=large entomological cages with larvae) are shown for gravid *Ae. aegypti* and for gravid *Ae. albopictus* in (**C**,**D**) in that order. Results of Experiment 3 (=large entomological cages without larvae) are shown for gravid *Ae. aegypti* and for gravid *Ae. albopictus* in (**E**,**F**) in that order.

**Figure 3:**
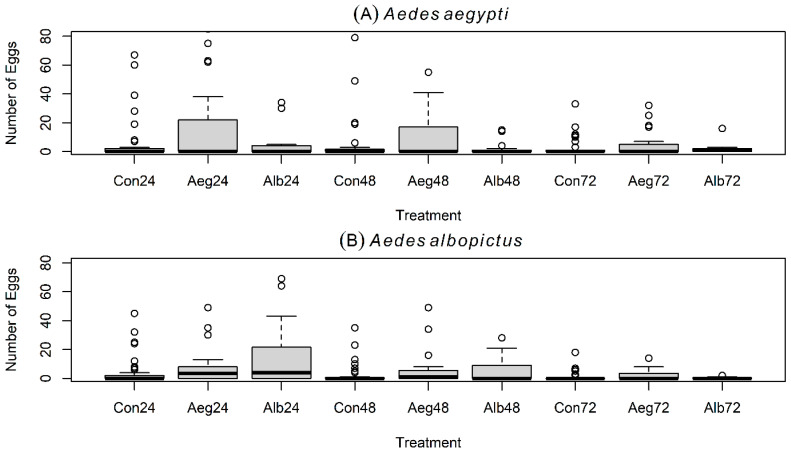
Boxplots (median and quantiles) of eggs laid by gravid *Aedes* spp. mosquitoes in experiment # 1. Results are shown for gravid females of *Ae. aegypti* (**A**) and *Ae. albopictus* (**B**) in small trial cages (i.e., 30 × 30 × 30 cm) and with 4 oviposition choices in total: two treatments (i.e., 20 conspecific larvae or 20 heterospecific larvae per treatment) and two controls (i.e., 2 oviposition cups containing water). Con = Control at 24, 48, and 72 h; Aeg = *Aedes aegypti* at 24, 48, and 72 h; Alb = *Aedes albopictus* at 24, 48, and 72 h.

In the first experiment with small cages, two treatment cups (i.e., 20 conspecific L4-larvae versus 20 heterospecific L4-larvae per treatment), and two controls (i.e., cups with only filtered dechlorinated tap water) (Figure 1B), gravid *Ae. aegypti* oviposited a greater average number of eggs in containers with conspecific larvae, compared with containers holding *Ae. albopictus* larvae or controls (Table 1; Figure 2A). The largest number of eggs in this experiment was laid during the first 24 h (Figure 3A). Results from the GLMMs indicated that the number of eggs laid by *Ae. aegypti* is positively and significantly predicted by the presence of conspecific L4-larvae (Z  =  11.987; *p*  <  0.001), but negatively and significantly predicted by the presence of *Ae. albopictus*’ L4-larvae (Z  =  −2.52; *p*  <  0.05), oviposition time at 48 h (Z  =  −2.701; *p*  <  0.01), oviposition time at 72 h (Z  =  −7.439; *p*  <  0.001), and by the interaction between the presence of *Ae. albopictus*’ L4-larvae and oviposition time at 48 h (Z  =  −2.827; *p*  <  0.01) (Table 2).

### 3.2. Oviposition Experiment # 1 with Gravid Aedes albopictus

Comparably, under the same trial scheme of experiment 1, gravid *Ae. albopictus* oviposited a greater average number of eggs in containers with conspecific L4-larvae, compared with controls. However, *Ae. albopictus* also laid a considerable number of eggs in containers holding *Ae. aegypti* L4-larvae (Table 1; Figure 2B). Moreover, *Ae. albopictus* laid the largest number of eggs during the first 24 h (Figure 3B). Results from the GLMMs revealed that the number of eggs laid by *Ae. albopictus* is positively and significantly predicted by the presence of both conspecific L4-larvae (Z  =  12.561; *p*  <  0.001) and heterospecific L4-larvae (Z  =  6.263; *p*  <  0.001), while being negatively and significantly predicted by oviposition time at 48 h (Z  =  −4.172; *p*  <  0.001), oviposition time at 72 h (Z  =  −7.522; *p*  <  0.001), and the interaction between the presence of conspecific larvae and oviposition time at 48 h (Z  =  −2.95; *p*  <  0.01) and at 72 h (Z  =  −6.069; *p*  <  0.001) (Table 3).

### 3.3. Oviposition Experiment # 2 with Gravid Aedes aegypti

**Figure 4:**
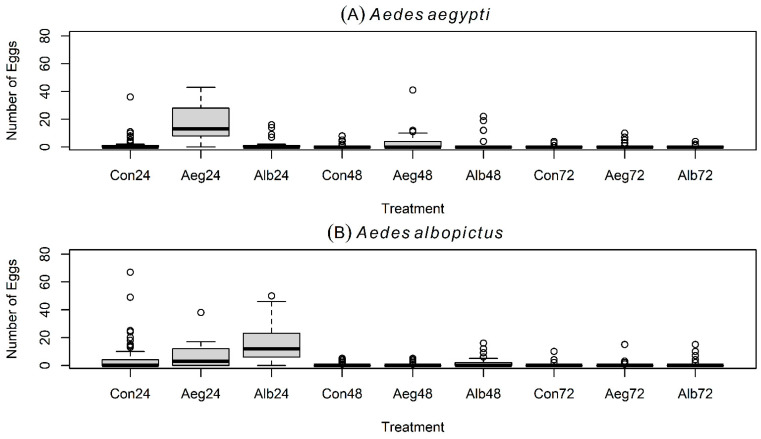
Boxplots (median and quantiles) of eggs laid by gravid *Aedes* spp. mosquitoes in experiment # 2. Results are shown for gravid females of *Ae. aegypti* (**A**) and *Ae. albopictus* (**B**) in large trial cages and with 6 oviposition choices in total: two treatments (i.e., 100 conspecific or 100 heterospecific larvae per treatment) and four controls (i.e., 4 oviposition cups containing only water). Con = Control at 24, 48, and 72 h; Aeg = *Aedes aegypti* at 24, 48, and 72 h; Alb = *Aedes albopictus* at 24, 48, and 72 h.

In the second experiment with large cages, two treatment cups (i.e., 100 conspecific L4-larvae versus 100 heterospecific L4-larvae per treatment), and four controls (i.e., cups with filtered dechlorinated tap water) (Figure 1B), gravid *Ae. aegypti* oviposited a greater average number of eggs in containers with conspecific L4-larvae, compared with containers holding *Ae. albopictus*’ L4-larvae and controls (Table 1; Figure 2C). The largest number of eggs was laid during the first 24 h (Figure 4A). Results from the GLMMs indicated that the number of eggs laid by *Ae. aegypti* is positively and significantly predicted by the presence of conspecific L4-larvae (Z  =  28.277; *p*  <  0.001), but also by the presence of heterospecific L4-larvae (Z  =  2.64; *p*  <  0.01), the interaction between the presence of heterospecific L4-larvae and oviposition times at 48 h (Z  =  5.831; *p*  <  0.001) and the interaction between the presence of heterospecific L4-larvae and oviposition times at 72 h (Z  =  2.462; *p*  <  0.05) (Table 2). In addition, the number of eggs laid by *Ae. aegypti* is negatively and significantly predicted by oviposition time at 48 h (Z  =  −7.26; *p*  <  0.001), and oviposition time at 72 h (Z  =  −8.072; *p*  <  0.001) (Table 2).

### 3.4. Oviposition Experiment # 2 with Gravid Aedes albopictus

Comparably, under the same trial scheme of experiment 2, gravid *Ae. albopictus* distributed eggs more evenly between conspecific and heterospecific containers while also laying fewer eggs in controls (Table 1; Figure 2D). The largest number of eggs was again laid during the first 24 h of the experiment (Figure 4B). Results from the GLMMs showed that the number of eggs laid by *Ae. albopictus* is positively and significantly predicted by the presence of both conspecific L4-larvae (Z  =  20.953; *p*  <  0.001) and heterospecific L4-larvae (Z  =  6.252; *p*  <  0.001), the interaction between the presence of conspecific L4-larvae and oviposition time at 48 h (Z  =  3.216; *p*  <  0.01) and 72 h (Z  =  2.562; *p*  <  0.05), and the interaction between the presence of heterospecific L4-larvae and oviposition time at 48 h (Z  =  3.040; *p*  <  0.01) (Table 3). In addition, the number of eggs laid by *Ae. albopictus* is negatively and significantly predicted by oviposition time at 48 h (Z  =  −14.254; *p*  <  0.01), and oviposition time at 72 h (Z  =  −13.496; *p*  <  0.001) (Table 3).

### 3.5. Oviposition Experiment # 3 with Gravid Aedes aegypti

**Figure 5:**
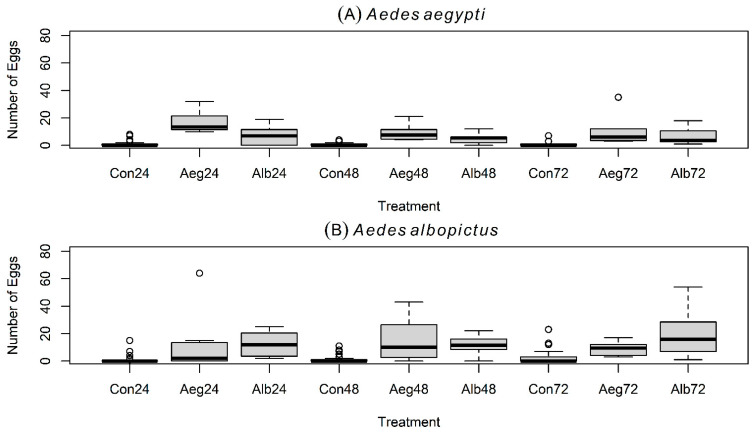
Boxplots (median and quantiles) of eggs laid by gravid *Aedes* spp. mosquitoes in experiment # 3. Results for gravid females of *Ae. aegypti* (**A**) and *Ae. albopictus* (**B**) in large trial cages and with 6 oviposition choices in total: either conspecific or heterospecific cups from experiment #2, in which 100 larvae were removed and four oviposition cups with only water. Con = Control at 24, 48, and 72 h; Aeg = *Aedes aegypti* at 24, 48, and 72 h; Alb = *Aedes albopictus* at 24, 48, and 72 h.

In the third experiment with large cages, four controls (i.e., cups with only dechlorinated tap water), and two treatments holding water that previously contained either 100 L4-larvae of *Ae. aegypti (i.e., conspecific* water—conditioned containers) or 100 L4-larvae of *Ae. albopictus (i.e., heterospecific* water—conditioned containers) (Figure 1C), gravid *Ae. aegypti* continued to oviposit a greater average number of eggs in conspecific L4-larvae water-conditioned containers (Table 1; Figure 2E). However, the number of eggs oviposited in containers where *Ae. albopictus* L4-larvae were previously situated also increased during this experiment. Moreover, egg counts also increased during the 48 h and 72 h, in both conspecific and heterospecific containers (Figure 5A). Results from the GLMMs indicated that the number of eggs laid by *Ae. aegypti* is positively and significantly predicted by the presence of both conspecific L4-larvae (Z  =  21.197; *p*  <  0.001) and heterospecific L4-larvae (Z  =  14.707; *p*  <  0.001), while being negatively and significantly predicted by oviposition time at 48 h (Z  =  −2.070; *p*  <  0.05) (Table 2).

### 3.6. Oviposition Experiment # 3 with Gravid Aedes albopictus

Comparably, under the same trial scheme of experiment 3, gravid *Ae. albopictus* continued to oviposit a uniform number of eggs in containers where conspecific and heterospecific L4-larvae were previously positioned (Table 1; Figure 2F). Also, the number of eggs laid by *Ae. albopictus* increased in heterospecific water-conditioned containers, and more eggs were also laid at 48 h and 72 h (Figure 5B). Results from the GLMMs showed that the number of eggs laid by *Ae. albopictus* is positively and significantly predicted by both conspecific L4-larvae water-conditioned containers (Z  =  12.182; *p*  <  0.001) and heterospecific L4-larvae water conditioned containers (Z  =  12.445; *p*  <  0.001), as well as by oviposition time at 72 h (Z  =  3.098; *p*  <  0.01), while being negatively and significantly predicted by the interaction between heterospecific L4-larvae water-conditioned containers and oviposition time at 72 h (Z  =  −4.400; *p*  <  0.001) (Table 3).

## 4. Discussion

### 4.1. Spatial–Temporal Oviposition Performances of Aedes aegypti and Aedes albopictus

During the first two experiments, with L4 larvae in treatment cups as visual and olfactory cues but different cage sizes, number of cups, and number of larvae per cup (Figure 1A,B), gravid *Ae. aegypti* oviposited a larger egg count in containers with conspecific L4 larvae compared with containers holding *Ae. albopictus* L4 larvae or controls. Similarly, in the third experiment, with only olfactory cues in water from where L4-larvae were removed, *Ae. aegypti* continued to oviposit a greater egg count in conspecific choices (Table 1; Figure 2A,C). However, the number of eggs oviposited in heterospecific water-conditioned containers also increased during the third experiment as compared with the first and second experiments (Figure 2E). Overall, these results suggest that both visual and olfactory cues are employed by *Ae. aegypti* to recognize conspecific L4 larvae and to avoid laying eggs in cups where L4 larvae of a competitor are located. The ability of *Ae. aegypti* to spatially discriminate the L4 larvae of its own species decreases markedly when visual signals are removed. This may indicate that, in our experimental design, visual cues play a more important role than olfactory cues in driving segregated oviposition in the presence of a competitor (Figure 5A). However, because we did not test the role of visual signals alone, our results more cautiously indicate that olfactory cues alone are insufficient to explain the oviposition segregation observed when both visual and chemical cues were present. Alternatively, olfactory cues could be important but short-lived, and therefore absent in “larva-conditioned” water once larvae were no longer actively present. Taken together, these findings imply that *Ae. aegypti* avoids ovipositing in containers holding heterospecific antagonist L4-larvae and in containers without any larvae (i.e., controls). In contrast to *Ae. aegypti*, gravid *Ae. albopictus* distributed eggs more evenly between conspecific and heterospecific options in the first and second experiments (Table 1; Figure 2B,D), and maintained this flexible oviposition performance even in the absence of visual cues during the third experiment (Figure 2F). These results suggest that *Ae. albopictus* lacks the heterospecific oviposition avoidance observed in *Ae. aegypti*.

*Aedes aegypti* and *Ae. albopictus* showed similar temporal oviposition performance during the first two experiments (Figure 1A,B): both produced higher egg counts within the first 24 h (Figure 3A,B and Figure 4A,B), and GLMM results revealed that egg counts were negatively and significantly predicted by oviposition times at 48 h and at 72 h in both species (Table 2 and Table 3). Furthermore, during the third experiment with only olfactory cues in water cups from which L4-larvae were removed, both mosquito species distributed eggs more evenly across time, increasing egg counts at 48 h and 72 h (Figure 5A,B). These outcomes may reflect decreased capacity of both species to discriminate between conspecific- and heterospecific-laden oviposition sites in the absence of visual signals, resulting in egg retention and delayed oviposition during trials. We observed no statistically significant differences in the mean CO_2_ production between the L4-larvae of *Ae. aegypti* and the L4-larvae of *Ae. albopictus*, suggesting that CO_2_ alone is unlikely to serve as a discriminatory non-visual signal to gravid females under a species coexistence scenario. Overall, our results suggest that CO_2_ or other persistent olfactory cues (present ≥1 h after larval removal) are unlikely to mediate the preference of *Ae. aegypti* for oviposition in conspecific-bearing containers. These cues, however, may mediate the observed preference for oviposition in occupied (heterospecific or conspecific) cups over pure water in both mosquito species. Instead, short-lived olfactory cues (present only ≤1 h after larval removal) or visual signals (e.g., the presence of conspecific or heterospecific L4-larvae in cups) may be essential for mediating the preference of *Ae. aegypti* for oviposition in conspecific-bearing containers.

To date, the oviposition performance of *Ae. albopictus* has been subject to less study than that of *Ae. aegypti*, and the oviposition behavior of these two vectors under experimental L4-larval co-occurrence scenarios has seldom been examined. Our results generally align with previous studies showing that gravid *Ae. aegypti* lay more eggs in conspecific-containing sites compared with controls [23]. Likewise, gravid *Ae. albopictus* typically exhibit flexible oviposition behavior, distributing eggs more evenly among available sites, particularly when a higher proportion of high-quality (i.e., undercrowded) larval habitats are available [11,12] or when paper lining is offered as an oviposition substrate [40]. Moreover, both *Ae. aegypti* and *Ae. albopictus* laid more eggs in water previously containing conspecific or heterospecific larvae than in controls [20,26], supporting the view that olfactory cues—whether bacterial or host-derived (i.e., volatile organic compounds)—contribute to site attractiveness [9,33]. The robust preference of both species for oviposition in previously larva-occupied water indicates the existence of oviposition-stimulating compounds in this water. Nevertheless, differences in oviposition performances under varying larval densities suggest that the species’ optimal oviposition strategies are density-dependent, and our results should therefore be interpreted in the context of the three co-occurrence experimental scenarios we tested [18,19].

### 4.2. Favorite Cup Versus Skip Oviposition

The spatio-temporal oviposition pattern of gravid *Ae. aegypti* and *Ae. albopictus* across three experiments suggests that, under an experimental co-occurrence scenario, there are idiosyncratic oviposition predispositions between these two species. Gravid *Ae. aegypti* avoids ovipositing where L4 larvae of its direct competitor are already located, while at the same time avoiding laying eggs at control sites lacking larvae. These results do not support the “Skip Oviposition” behavior anticipated for *Ae. aegypti* but instead suggest a “Favorite Cup” tendency, as the majority of eggs across experiments were laid in one conspecific container (Figure 2A,C,E). In contrast, gravid *Ae. albopictus* are more flexible than *Ae. aegypti*, ovipositing considerable egg counts in both conspecific and heterospecific choices while also avoiding laying eggs in controls (Figure 2B,D,F). Unlike *Ae. aegypti*, findings from *Ae. albopictus* do not support the “Favorite Cup” tendency, but rather, they provide support for the “Skip Oviposition” behavioral pattern. However, under an experimental co-occurrence scenario, this support is only partial, as controls were poor predictors of egg counts for *Ae. albopictus* in all three experiments (Table 2 and Table 3).

Our results show that neither *Ae. aegypti* nor *Ae. albopictus* strictly follow the “Favorite Cup” or “Skip Oviposition” tendencies. Instead, the oviposition performance of these two mosquito vectors in the presence of an antagonist species is a species-specific response to rapidly look for and identify containers where members of its own species are located (i.e., gravid *Ae. aegypti*), conceivably trying to avoid the interaction with a direct larval competitor, or equally favoring breeding sites with mosquito larvae present regardless of whether these are conspecifics or heterospecific (i.e., gravid *Ae. albopictus*). In the former case, gravid females may conceivably be trying to avoid interactions between their offspring and direct larval competitors; in the latter case, gravid females may conceivably be trying to increase the odds for interspecific larval competition for space and food resources. In fact, it seems that both mosquito species aggregate their egg batches after visually and chemically sensing the presence of conspecific and heterospecific L4 larvae. Our outcomes were consistent for both vector species across three discrete experimental settings, including an increasing number of containers, larval densities, and cage sizes, which indicates that oviposition phenomena under a scenario of species coexistence are a function of the species recognition capacity and the synergy between variation in space and time.

### 4.3. Implications for the Control of Aedes aegypti in Panama

Several biological mechanisms have been proposed to explain changes in the spatial demography of coexisting *Ae. aegypti* and *Ae. albopictus* mosquitoes, with interspecific larval competition and mating interference being the two most empirically supported up until now [6,7,58]. Other mechanisms, such as oviposition aggregation or segregation, have not been studied in detail under an experimental co-occurrence scenario. Our results suggest that a hypothetical scenario of interspecific larval competition is less likely if gravid *Ae. aegypti* encounters containers where *Ae. albopictus* L4 larvae are already present. The segregated conspecific oviposition performance of *Ae. aegypti* is expected to reduce the opportunities for *Ae. aegypti* larvae to interact with *Ae. albopictus*’ larvae, especially during the rainy season when a greater number of breeding containers might occur, and consequently, *Ae. aegypti* would have more chances to escape from larval competition. Interspecific larval competition, on the contrary, is likely to occur when gravid *Ae. albopictus* encounters containers where *Ae. aegypti* L4 larvae are already present. The flexible oviposition performance of *Ae. albopictus* is expected to increase the opportunities for their larvae to interact with *Ae. aegypti* larvae, particularly during the dry season when fewer breeding containers might occur, and thus, *Ae. aegypti* would have fewer chances to escape from larval competition. Both scenarios can have a negative impact on the local demography of *Ae. aegypti* by increasing mortality and reducing its effective population size. For example, in the presence of *Ae. albopictus* larvae, gravid *Ae. aegypti* might retain eggs and fly longer distances for extended time periods while searching for optimal breeding sites [27,59]. This can result in higher energy costs and greater female and egg mortality due to harsh environmental conditions or aerial and terrestrial predators. In the presence of *Ae. aegypti* L4 larvae, gravid *Ae. albopictus* deliberately lay eggs in heterospecific containers, increasing *Ae. aegypti*’s larval mortality via interspecific larval competition [6,58]. We posit that *Ae. aegypti* begins to interact with *Ae. albopictus* even before their larvae are set to compete for food resources within a particular breeding container. We also speculate that the segregated oviposition performance of gravid *Ae. aegypti* in the presence of a competitor, along with interspecific larval competition and mating interference with *Ae. albopictus*, is another factor contributing to its rapid displacement from certain areas of Panama [15,16].

Our findings have important ramifications for improving vector control strategies and reducing the health hazards associated with dengue and other *Aedes*-borne diseases in Panama. The development of more targeted vector control tactics in different regions of the country would be possible by identifying the relationship between container availability and the oviposition behavior of local populations of *Ae. aegypti* and *Ae. albopictus*. Interventions that limit mosquito access to artificial containers can reduce breeding opportunities for *Ae. aegypti* while allowing *Ae. albopictus* to displace this species, the most efficient dengue transmitter in Panama. Our results suggest that waste management must be improved, since a greater abundance of containers would likely enable *Ae. aegypti* to escape larval competition with *Ae. albopictus* and thereby increase dengue transmission in densely populated areas of Panama [48,49]. For example, improved management of the many used tires traded in garages along the country’s main highway system may affect *Ae. aegypti* by fostering interspecific larval competition with *Ae. albopictus* in the vicinity of these widely distributed businesses and by reducing opportunities for *Ae. aegypti* to colonize new areas via long-distance, human-assisted migration [50]. Our results reinforce how pivotal the role of container availability is on the oviposition behaviors of *Aedes* vectors, which is often the defining factor in their spatial and temporal demography as well as in the risk of disease transmission to humans. Panamanian health authorities should prioritize effective trash management and container reduction practices over the current reactive *Aedes* control measures, which rely almost exclusively on the use of synthetic insecticides to exterminate adult mosquitoes [60]. A targeted education campaign regarding trash management combined with community engagement in dengue endemic areas will foster a more effective and sustainable arbovirus mitigation approach in Panama.

## 5. Conclusions

Under experimental L4-larval co-occurrence scenarios, *Ae. aegypti* promptly locates and lay eggs in containers with L4-larvae of its own species, possibly to reduce the risk of larval competition with *Ae. albopictus*. The ability of *Ae. aegypti* to distinguish among sites bearing conspecific versus heterospecific L4 larvae, however, tends to decrease in the absence of visual signals. This leads to increased oviposition alongside heterospecifics, increasing the likelihood of interspecific larval competition. Contrary to *Ae. aegypti*, gravid *Ae. albopictus* lacks heterospecific oviposition avoidance behavior, showing no preference between conspecific or heterospecific-containing oviposition site alternatives. The combined effects of *Ae. aegypti*’s segregated oviposition performance and *Ae. albopictus*’s opportunistic oviposition behavior might increase *Ae. aegypti* larval mortality, negatively impacting populations in sympatry and partly explaining their rapid displacement from some areas of Panama. Future studies should test our results using a semi-experimental design in which additional treatment combinations can be evaluated (e.g., L1-L3 larval stages and other mosquito species), advanced methods for analyzing volatile organic compounds can be applied, and greater biological complexity in oviposition behavior (e.g., larger distances between oviposition cups) can be incorporated.

## Figures and Tables

**Table 1:** Result summary from three oviposition experiments simulating co-occurrence scenarios between gravid *Aedes aegypti* and gravid *Aedes albopictus* mosquitoes. The total number of eggs laid by *Aedes* Stegomyia mosquitoes and (The mean number of eggs and the standard deviation) are shown for both *Aedes aegypti* and *Aedes albopictus* in their respective treatment category: L4-larvae conspecific, L4-larvae heterospecific, and controls. In the column for experiment, *n* indicates, respectively, the total number of cups for the control, conspecific, and heterospecific treatments.

Experiment	*Aedes* Stegomyia *aegypti*			*Aedes* Stegomyia *albopictus*		
	Control	Conspecific	Heterospecific	Control	Conspecific	Heterospecific
# 1 (*n* = 120, 60, 60)	531 (4.43 ± 12.93)	635 (10.58 ± 20.63)	161 (2.68 ± 6.47)	320 (2.67 ± 7.30)	394 (6.57 ± 14.27)	340 (5.67 ± 11.17)
# 2 (*n* = 348, 87, 87)	189 (0.54 ± 2.38)	636 (7.31 ± 11.41)	130 (1.49 ± 4.13)	499 (1.43 ± 5.52)	549 (6.31 ± 10.70)	231 (2.66 ± 5.78)
# 3 (*n* = 96, 24, 24)	72 (0.75 ± 1.56)	288 (12.00 ± 8.89)	147 (6.13 ± 5.68)	146 (1.52 ± 3.72)	351 (14.63 ± 11.93)	287 (11.96 ± 15.34)

**Table 2:** Poisson Generalized Linear Mixed Model (GLMMs) results of the response variable “egg counts” for gravid *Aedes* Stegomyia *aegypti* and predictors (e.g., Larvae Conspecific versus Larvae Heterospecific), time (e.g., 24, 48, and 72 h), and their interaction in three experiments under a co-occurrence scenario. Signif. Codes: 0 ‘***’ 0.001 ‘**’ 0.01 ‘*’ 0.05.

Experiment # 1				
Fixed effects	Estimate	Std. Error	z value	Pr (>|z|)
Intercept	1.7169	0.112	15.291	<2 × 10^−16^ ***
Larvae (Conspecific)	1.008	0.084	11.987	<2 × 10^−16^ ***
Larvae (Heterospecific)	−0.311	0.123	−2.520	0.01173 *
Time at 48 h.	−0.261	0.096	−2.701	0.00691 **
Time at 72 h.	−0.877	0.118	−7.439	1.02 × 10^−13^ ***
Larvae (Conspecific) × 48 h.	−0.257	0.131	−1.955	0.05059
Larvae (Heterospecific) × 48 h.	−0.615	0.217	−2.827	0.00469 **
Larvae (Conspecific) × 72 h.	−0.285	0.162	−1.757	0.07897
Larvae (Heterospecific) × 72 h.	−0.055	0.231	−0.239	0.81093
Random effects	Variance	Std. Dev.		AIC
Assay	0.1676	0.4094		4198.0
Experiment # 2				
Fixed effects	Estimate	Std. Error	z value	Pr (>|z|)
Intercept	0.064	0.128	0.504	0.6143
Larvae (Conspecific)	2.666	0.094	28.277	<2 × 10^−16^ ***
Larvae (Heterospecific)	0.419	0.158	2.640	0.0083 **
Time at 48 h.	−1.318	0.181	−7.260	3.88 × 10^−13^ ***
Time at 72 h.	−2.758	0.341	−8.072	6.90 × 10^−16^ ***
Larvae (Conspecific) × 48 h.	−0.323	0.211	−1.525	0.1273
Larvae (Heterospecific) × 48 h.	1.503	0.257	5.831	5.50 × 10^−9^ ***
Larvae (Conspecific) × 72 h.	−0.219	0.395	−0.554	0.5793
Larvae (Heterospecific) × 72 h.	1.167	0.474	2.462	0.0138 *
Random effects	Variance	Std. Dev.		AIC
Assay	0.266	0.516		2081.3
Experiment # 3				
Fixed effects	Estimate	Std. Error	z value	Pr (>|z|)
Intercept	−0.094	0.207	−0.457	0.6473
Water (Conspecific)	2.772	0.130	21.197	<2 × 10^−16^ ***
Water (Heterospecific)	2.101	0.142	14.707	<2 × 10^−16^ ***
Time at 48 h.	−0.529	0.255	−2.070	0.0385 *
Time at 72 h.	−0.461	0.255	−1.807	0.0707
Random effects	Variance	Std. Dev.		AIC
Assay	0.208	0.456		621.5

**Table 3:** Poisson Generalized Linear Mixed Model (GLMMs) results of the response variable “egg counts” for gravid *Aedes* Stegomyia *albopictus* and predictors (e.g., Larvae Conspecific versus Larvae Heterospecific), time (e.g., 24, 48, and 72 h), and their interaction in three experiments under a co-occurrence scenario. Signif. Codes: 0 ‘***’ 0.001 ‘**’ 0.01 ‘*’ 0.05.

Experiment # 1				
Fixed effects	Estimate	Std. Error	z value	Pr (>|z|)
Intercept	1.401	0.101	13.869	<2 × 10^−16^ ***
Larvae (Heterospecific)	0.681	0.108	6.263	3.78 × 10^−10^ ***
Larvae (Conspecific)	1.215	0.096	12.561	<2 × 10^−16^ ***
Time at 48 h.	−0.524	0.125	−4.172	3.02 × 10^−5^ ***
Time at 72 h.	−1.198	0.159	−7.522	5.38 × 10^−14^ ***
Larvae (Heterospecific) × 48 h.	0.266	0.171	1.553	0.12051
Larvae (Conspecific) × 48 h.	−0.502	0.170	−2.950	0.00318 **
Larvae (Heterospecific) × 72 h.	−0.135	0.232	−0.585	0.55881
Larvae (Conspecific) × 72 h.	−2.508	0.413	−6.069	1.29 × 10^−9^ ***
Random effects	Variance	Std. Dev.		AIC
Assay	0.084	0.2911		2950.9
Experiment # 2				
Fixed effects	Estimate	Std. Error	z value	Pr (>|z|)
Intercept	1.286	0.088	14.547	<2 × 10^−16^ ***
Larvae (Heterospecific)	0.533	0.085	6.252	4.05 × 10^−10^ ***
Larvae (Conspecific)	1.384	0.066	20.953	<2 × 10^−16^ ***
Time at 48 h.	−2.862	0.200	−14.254	<2 × 10^−16^ ***
Time at 72 h.	−3.229	0.239	−13.496	<2 × 10^−16^ ***
Larvae (Heterospecific) × 48 h.	0.366	0.327	1.120	0.26288
Larvae (Conspecific) × 48 h.	0.787	0.244	3.216	0.00130 **
Larvae (Heterospecific) × 72 h.	1.006	0.331	3.041	0.00236 **
Larvae (Conspecific) × 72 h.	0.749	0.292	2.562	0.01040 *
Random effects	Variance	Std. Dev.		AIC
Assay	0.159	0.399		2975.1
Experiment # 3				
Fixed effects	Estimate	Std. Error	z value	Pr (>|z|)
Intercept	−0.128	0.224	−0.571	0.5687
Water (Heterospecific)	2.506	0.205	12.182	<2 × 10^−16^ ***
Larvae (Conspecific)	2.547	0.204	12.445	<2 × 10^−16^ ***
Time at 48 h.	0.331	0.304	1.088	0.27679
Time at 72 h.	0.887	0.286	3.098	0.00195 **
Water (Heterospecific) × 48 h.	−0.037	0.273	−0.138	0.89025
Water (Conspecific) × 48 h.	−0.331	−0.331	−1.198	0.23111
Water (Heterospecific) × 72 h.	−1.161	0.264	−4.401	1.08 × 10^−5^ ***
Water (Conspecific) × 72 h.	−0.402	0.2481	−1.623	0.10467
Random effects	Variance	Std. Dev.		AIC
Assay	0.143	0.378		1227.0

## Data Availability

The original contributions presented in this study are included in the article/Supplementary Materials. Further inquiries can be directed to the corresponding author(s).

## Supplementary Materials

The following supporting information can be downloaded at: https://www.mdpi.com/article/10.3390/insects16111110/s1, Figure S1: One-way Analysis of Variance (ANOVA) for the mean CO_2_ production comparison among L4-larvae *Aedes aegypti*, L4-larvae *Aedes albopictus*, and control.

## Abbreviations

The following abbreviations are used in this manuscript:

Bti	*Bacillus thuringiensis israelensis*
AP	Azuero Peninsula
GLMM	Generalized Linear Mixed Effects Models
AIC	Akaike Information Criterion
ANOVA	Analysis of Variance
HSD	Post Hoc Tukey’s
Aeg	*Aedes aegypti*
Alb	*Aedes albopictus*
Con	Control
INDICASAT	Instituto de Investigaciones Científicas y Servicios de Alta Tecnología
SENACYT	Secretariat for Science, Technology, and Innovation
STRI	Smithsonian Tropical Research Institute
UP	University of Panama
SNI	National System of Investigation
