# Supplementary material for: Oviposition Behavior of Aedes aegypti and Aedes albopictus (Diptera: Culicidae) from Panama Under Experimental L4-Larval Co-Occurrence Scenarios
Source: Insects. 2025 Oct 31;16(11):1110. doi: 10.3390/insects16111110 (PMC12653154; doi:10.3390/insects16111110)
Supplement: Supplementary file 1 [file insects-16-01110-s001.zip › insects-3834366-supplementary.pdf]

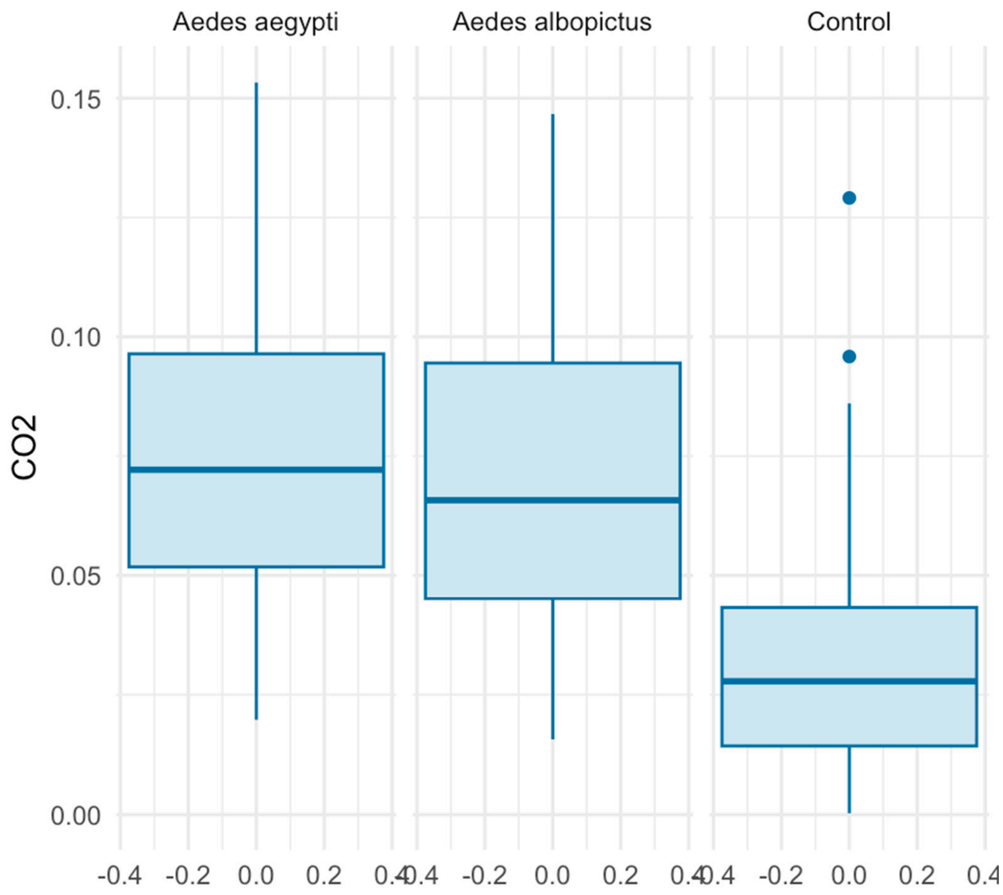

Supplementary Figure S1. One-way Analysis of Variance (ANOVA) for the mean CO_2 production comparison among L4-larvae *Aedes aegypti*, L4-larvae *Aedes albopictus*, and control.
